# Prediction model development of late-onset preeclampsia using machine learning-based methods

**DOI:** 10.1371/journal.pone.0221202

**Published:** 2019-08-23

**Authors:** Jong Hyun Jhee, SungHee Lee, Yejin Park, Sang Eun Lee, Young Ah Kim, Shin-Wook Kang, Ja-Young Kwon, Jung Tak Park

**Affiliations:** 1 Division of Nephrology, Department of Internal Medicine, Gangnam Severance Hospital, Yonsei University College of Medicine, Seoul, Korea; 2 Department of Internal Medicine, College of Medicine, Institute of Kidney Disease Research, Yonsei University, Seoul, Korea; 3 Department of Preventive Medicine, Yonsei University College of Medicine, Seoul, Korea; 4 Biostatics Collaboration Unit, Yonsei University College of Medicine, Seoul, Korea; 5 Division of Maternal-Fetal Medicine, Institute of Women’s Medical Life Science, Department of Obstetrics and Gynecology, Yonsei University College of Medicine, Seoul, Korea; 6 Department of Medical Informatics, Yonsei University Health System, Seoul, Korea; University of Mississippi Medical Center, UNITED STATES

## Abstract

Preeclampsia is one of the leading causes of maternal and fetal morbidity and mortality. Due to the lack of effective preventive measures, its prediction is essential to its prompt management. This study aimed to develop models using machine learning to predict late-onset preeclampsia using hospital electronic medical record data. The performance of the machine learning based models and models using conventional statistical methods were also compared. A total of 11,006 pregnant women who received antenatal care at Yonsei University Hospital were included. Maternal data were retrieved from electronic medical records during the early second trimester to 34 weeks. The prediction outcome was late-onset preeclampsia occurrence after 34 weeks’ gestation. Pattern recognition and cluster analysis were used to select the parameters included in the prediction models. Logistic regression, decision tree model, naïve Bayes classification, support vector machine, random forest algorithm, and stochastic gradient boosting method were used to construct the prediction models. C-statistics was used to assess the performance of each model. The overall preeclampsia development rate was 4.7% (474 patients). Systolic blood pressure, serum blood urea nitrogen and creatinine levels, platelet counts, serum potassium level, white blood cell count, serum calcium level, and urinary protein were the most influential variables included in the prediction models. C-statistics for the decision tree model, naïve Bayes classification, support vector machine, random forest algorithm, stochastic gradient boosting method, and logistic regression models were 0.857, 0.776, 0.573, 0.894, 0.924, and 0.806, respectively. The stochastic gradient boosting model had the best prediction performance with an accuracy and false positive rate of 0.973 and 0.009, respectively. The combined use of maternal factors and common antenatal laboratory data of the early second trimester through early third trimester could effectively predict late-onset preeclampsia using machine learning algorithms. Future prospective studies are needed to verify the clinical applicability algorithms.

## Introduction

Preeclampsia, which affects 5–8% of pregnancies worldwide, is one of the leading causes of maternal and fetal morbidity and mortality [1–3]. Maternal complications associated with preeclampsia include placental abruption and acute kidney disease. In severe cases, preeclampsia leads to eclamptic seizures and life-threatening hemolysis, elevated liver enzymes, and low platelet count (HELLP) syndrome [4]. Fetal complications related to preeclampsia include impaired fetal growth, neonatal respiratory distress syndrome, and stillbirth. Preeclampsia can be classified as early-onset preeclampsia, which develops before 34 weeks’ gestation, and the more common late-onset preeclampsia, which develops at or after 34 weeks’ gestation [5].

Despite the serious clinical consequences, there is currently no effective preventive measure for preeclampsia. Close surveillance and early detection, which enable its prompt management, comprise the main clinical management strategy. Therefore, studies have focused on developing useful preeclampsia prediction methods [6]. A practical prediction model would allow increased surveillance of at-risk patients and reduce surveillance of patients who are less likely to develop preeclampsia. Although previous studies have analyzed clinical features and evaluated biomarkers for effective prediction, few have demonstrated clinically sufficient properties [7–11].

Machine learning (ML) techniques provide the possibility to infer significant connections between data items from diverse data sets that are otherwise difficult to correlate [12,13]. Due to the vast amount and complex nature of medical information, ML is recognized as a promising method for diagnosing diseases or predicting clinical outcomes. Several ML techniques have been applied in clinical settings and shown to predict diseases with higher accuracy than conventional methods [14,15].

The specific aims of this study were to develop models using ML to predict late-onset preeclampsia using hospital electronic medical record data and compare the performance of the models developed from ML and conventional statistical methods.

## Materials and methods

### Study population

This study included 11,006 pregnant women who received antenatal care at Yonsei University Healthcare Center (Severance hospital and Gangnam Severance hospital) in Seoul, Korea between 2005 and 2017. Patients with pregnancy termination prior to 24 weeks’ gestation due to miscarriage, fetal death, or early-onset preeclampsia or those who did not deliver at the Yonsei University Healthcare Center were excluded from the study. Antenatal care and evaluations were performed following common hospital protocols. The study protocol was approved by the institutional review board of Yonsei University Health System (4-2017-0096). Informed consent was waived by the institutional review boards owing to the retrospective study design.

### Clinical and biochemical data collection

Demographic and laboratory data during the antenatal period were retrieved from electronic medical records. Antenatal data were obtained for each individual repeatedly from the early second trimester to gestational age of 34 weeks. Gestational age 14–17 weeks was considered as early second trimester. The clinical data included age, blood pressure (BP), height, weight, and gestational age. Maternal medical history of hypertension, diabetes, and previous preeclampsia as well as obstetrical and social histories and medications prescribed during pregnancy were also retrieved. The following biochemical laboratory data were also collected: blood urea nitrogen (BUN), serum creatinine, spot urine protein to creatinine ratio (UPCR), urine albumin to creatinine ratio, hemoglobin, fasting blood glucose, serum albumin, uric acid, total bilirubin, aspartate transaminase (AST), alanine transaminase (ALT), total cholesterol, triglycerides, high-density lipoprotein cholesterol, and low-density lipoprotein cholesterol.

### Study outcome

The study endpoint was the development of late-onset preeclampsia defined as new-onset hypertension (diastolic BP ≥ 90 mm Hg or systolic BP ≥ 140 mm Hg measured on two occasions at least 2 hours apart) accompanied by clinically significant proteinuria defined as one of the following: random urine dipstick results of at least 1+ on two occasions or results of at least 2+; a 24-hour urine protein level ≥ 300 mg; or a platelet count <100,000/μL, creatinine level > 1.1 mg/dL, serum transaminase levels twice normal, or cerebral symptoms or pulmonary edema occurring after 34 weeks’ gestation [16].

### Selection of prediction model variables

**Fig 1:**
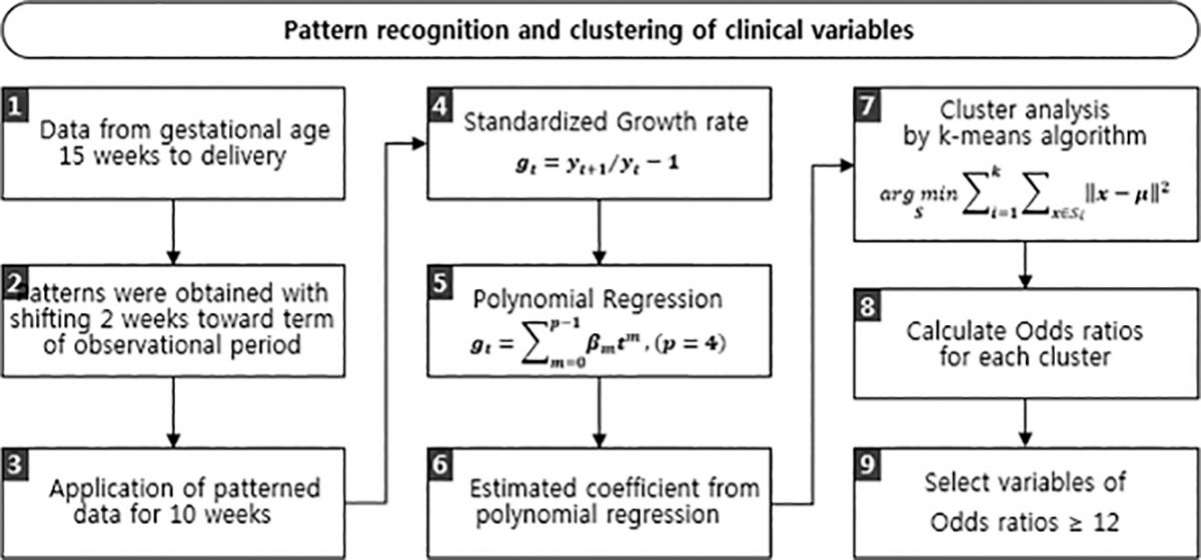
Flow chart of pattern recognition and cluster analysis based variable selection process for late-onset preeclampsia prediction.

For the repeated-measured data, such as BP, body weight, and laboratory data, significant variables to be included in the prediction models were delineated through pattern recognition and cluster analysis (Fig 1) [17,18]. Pattern recognition and cluster analysis allows use of the value of the variable itself and the changing pattern of the variable during the repeated measurement period as analysis factors. The changes in individual variables during each of the 10-week windows were patternized. Each window was shifted by a 2-week interval beginning from 14 weeks’ to 34 weeks’ gestational age. Subsequently, the patterned data were applied to the sequential polynomial regression analysis. From this polynomial regression, coefficients were estimated and used in the cluster analysis by the k-means algorithm. Odds ratios were calculated of each cluster. The variables with odds ratios > 12 were considered to have significant pattern changes during the antenatal period and selected for inclusion in the prediction models.

### Primary analysis

The individuals included in the study were randomly divided into training (70% of sample) and validation (30% of sample) sets [19]. Women who developed late-onset preeclampsia were categorized into the preeclampsia group, while those who did not develop preeclampsia were categorized into the no preeclampsia group. The characteristics at early second trimester were compared between the preeclampsia and no preeclampsia groups. The normality of the distribution was analyzed using the Kolomogorov-Smirnov test. Intergroup comparisons were performed using Student’s t-test for normally distributed continuous variables, while variables that did not show a normal distribution was compared using the Kruskal–Wallis test and presented as median with interquartile range. For clinically important candidate variables with missing data, multiple imputation was used, with 25 imputed data sets generated using fully conditional specification methods to generate the final estimates.

Six methods were used for prediction model development and compared. The repeated measured values of the variables selected from the pattern recognition and cluster analysis were used in the prediction models. These data included those from antenatal evaluations starting from early trimester until gestational age of 34 weeks. In addition to these repeated measured variables, non-repeated measured variables such as maternal medical history, obstetrical and social history, and medication prescription history during pregnancy were also included in the prediction models. The prediction outcome was late-onset preeclampsia occurrence after 34 weeks’ gestational age. The methods used for prediction model construction were logistic regression (LR), decision tree model (DT), naïve Bayes classification (NBC), support vector machine (SVM), random forest algorithm (RF), and stochastic gradient boosting method (SGB) [20–25]. For LR, variables were entered into the model by backward elimination. For RF, the number of decision trees was set to 250. The number of repetition boosts in the SGB was also set to 250. For RF and SGB, the number of variables to be sampled as split candidates in the nodes of each tree was defined as the number of √ variables = √85 ≒ 9. All prediction models were implemented using R programming language (software 3.3.1 (http://www.R-project.org).). To assess the relative importance of the selected variables in each prediction model, absolute t-score was used for the LR model, 1-accuracy for model excluding the relevant variable was used for the NBC model, and IncNodePurity was used in DT, SVM, RF, and SGB models.

Each model’s performance was evaluated and compared using the validation data set. The receiver operating characteristic curve and area under the curve were used to evaluate the model’s ability to predict late-onset preeclampsia. Model calibration was evaluated using plots of predicted vs. observed rates of preeclampsia development. C-statistics was used to assess the performance of each prediction model.

## Results

### Clinical characteristics

The clinical characteristics of study subjects obtained at early second trimester are shown in Table 1. Among the 11,006 enrolled individuals, preeclampsia was subsequently diagnosed in 474 (4.7%) women after 34 weeks’ of gestation. Subjects who developed preeclampsia were older than those who did not develop preeclampsia. Parity number did not differ between the two groups. Systolic and diastolic BP were both significantly higher in those who developed preeclampsia than in those who did not. Regarding maternal medical history, subjects who developed preeclampsia were more likely to have chronic hypertension and have been diagnosed with preeclampsia in previous pregnancies than those who did not develop preeclampsia. When laboratory test results were compared, UPCR, total bilirubin, AST, ALT, BUN, creatinine, and hemoglobin levels at early second trimester were higher but platelet count was lower in subjects who developed preeclampsia than in those who did not.

**Table 1:** Maternal characteristics and laboratory parameters at early second trimester.

	No preeclampsia (n = 10,058)	Preeclampsia (n = 474)	*P*
Maternal age, years	38.9 ± 5.0	44.1 ± 20.2	<0.001
Parity number	1.9 ± 1.1	2.0 ± 1.1	0.07
Height, cm	160.9 ± 7.1	159.8 ± 7.8	<0.001
Maternal weight at pregnancy, kg	57.8 ± 10.0	60.1 ± 11.8	<0.001
SBP, mmHg	111.73 ± 8.7	116.7 ± 12.3	<0.001
DBP, mmHg	67.8 ± 6.5	71.6 ± 9.2	<0.001
Maternal history, n (%)			
Smoking, n (%)	36 (0.3)	4 (0.9)	0.05
Alcohol, n (%)	108 (1.0)	7 (1.6)	0.26
Hypertension	154 (1.4)	75 (16.8)	<0.001
Diabetes	425 (4.0)	18 (4.0)	0.98
Preeclampsia	5 (0.1)	6 (1.3)	<0.001
Laboratory data			
WBC, 10^3^/uL	9.01 ± 4.17	11.04 ± 3.45	<0.001
Hemoglobin, g/dL	11.6 ± 1.4	13.5 ± 12.4	<0.001
Platelet counts, 10^9^/L	200.1 ± 57.0	195.5 ± 63.3	<0.001
BUN, mg/dL	5.7 ± 3.2	9.9 ± 8.2	<0.001
Creatinine, mg/dL	0.4 ± 0.2	0.7 ± 0.7	<0.001
Total bilirubin, mg/dL	0.3 ± 0.2	0.4 ± 0.4	<0.001
AST, IU/L	15.8 ± 18.4	24.8 ± 41.5	<0.001
ALT, IU/L	12.8 ± 16.7	20.0 ± 29.7	<0.001
Potassium, mEq/L	4.1 ± 0.3	4.3 ± 0.2	0.36
TCO_2,_ mEq/L	21.9 ± 2.1	20.6 ± 2.6	0.17
Calcium, mg/dL	8.5 ± 0.5	8.7 ± 1.1	0.43
Magnesium, mg/dL	1.2 ± 0.1	1.6 ± 0.0	0.56
UPCR, g/gCr	0.09 [0.02–0.12]	0.20 [0.08–0.26]	0.87

Data are presented as mean ± standard deviation or number (%)

***Abbreviation*:** SBP, systolic blood pressure; DBP, diastolic blood pressure; UPCR, urine protein to creatinine ratio

Table 2 summarizes the clinical characteristics of the study subjects at delivery. Maternal weight was higher and systolic and diastolic BP were significantly higher in women who developed preeclampsia than in those who did not. UPCR, AST, ALT, BUN, and serum creatinine levels were higher at delivery while platelet counts were lower in subjects who were diagnosed with preeclampsia than in those who were not.

**Table 2:** Clinical characteristics and laboratory parameters at delivery.

	No preeclampsia (n = 100,58)	Preeclampsia (n = 474)	*P*
Maternal weight at delivery, kg	62.8 ± 9.3	64.0 ± 10.9	<0.001
SBP, mmHg	113.5 ± 12.0	145.0 ± 22.6	<0.001
DBP, mmHg	68.4 ± 8.9	89.8 ± 15.4	<0.001
Laboratory data			
WBC, 10^3^/uL	9.4 ± 2.8	11.0 ± 4.7	<0.001
Hemoglobin, g/dL	11.8 ± 1.3	11.9 ± 1.8	0.15
Platelet counts, 10^9^/L	228.8 ± 63.9	207.3 ± 79.3	<0.001
BUN, mg/dL	7.8 ± 2.5	12.4 ± 5.3	<0.001
Creatinine, mg/dL	0.5 ± 0.2	0.7 ± 0.3	<0.001
Total bilirubin, mg/dL	0.5 ± 0.2	0.5 ± 0.8	0.51
AST, IU/L	18.3 ± 0.5	54.7 ± 20.7	<0.001
ALT, IU/L	12.9 ± 0.4	37.8 ± 9.9	<0.001
Potassium, mEq/L	4.1 ± 0.3	4.3 ± 0.3	<0.001
TCO_2,_ mEq/L	21.8 ± 2.2	20.9 ± 2.8	<0.001
Calcium, mg/dL	8.7 ± 0.4	8.4 ± 0.7	<0.001
Magnesium, mg/dL	1.5 ± 0.5	1.6 ± 0.7	0.85
UPCR, g/gCr	0.15 [0.08–0.21]	0.36 [0.20–0.40]	<0.001

Data are presented as mean ± standard deviation or number (%)

***Abbreviation*:** SBP, systolic blood pressure; DBP, diastolic blood pressure; UPCR, urine protein to creatinine ratio

### Variable influence on prediction

**Fig 2:**
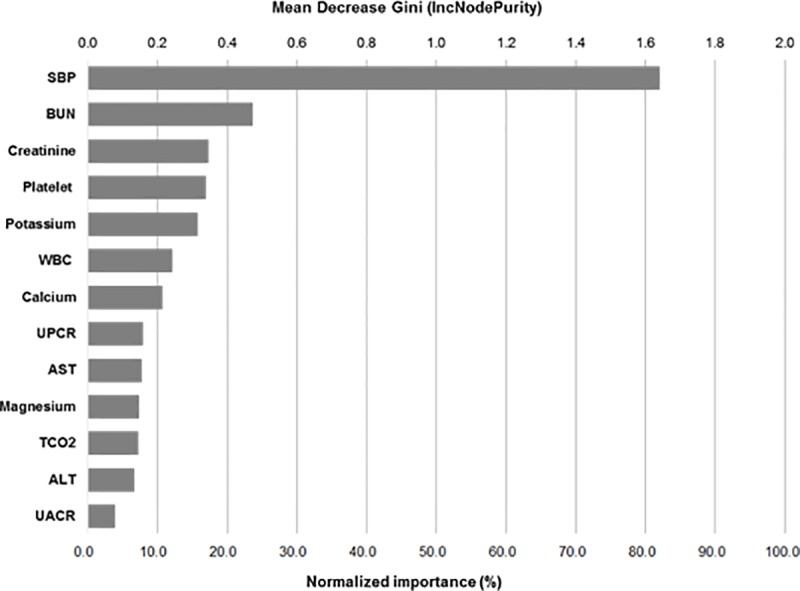
Normalized importance of the selected variables for late-onset preeclampsia prediction models. The plot shows relative importance of the variables in random forest model. IncNodePurity reflects the reduction in entropy, which is the uncertainty, due to sorting of the attribute. ***Abbreviation*:** SBP, systolic blood pressure; WBC, white blood cell; UPCR, urine protein to creatinine ratio; UACT, urine albumin to creatinine ratio.

Using the pattern recognition and cluster analysis, the influence of each variable on prediction was evaluated. Among the assessed variables, the 14 most influential factors were included in the prediction models. Systolic BP, followed by serum BUN and creatinine level, and platelet count were the most important variables. Interestingly, white blood cell count, serum calcium level, and serum magnesium level were also delineated as influential variables (Fig 2). The relative importance of the selected variables in each prediction model are described in S1 Table.

### Model performance

**Fig 3:**
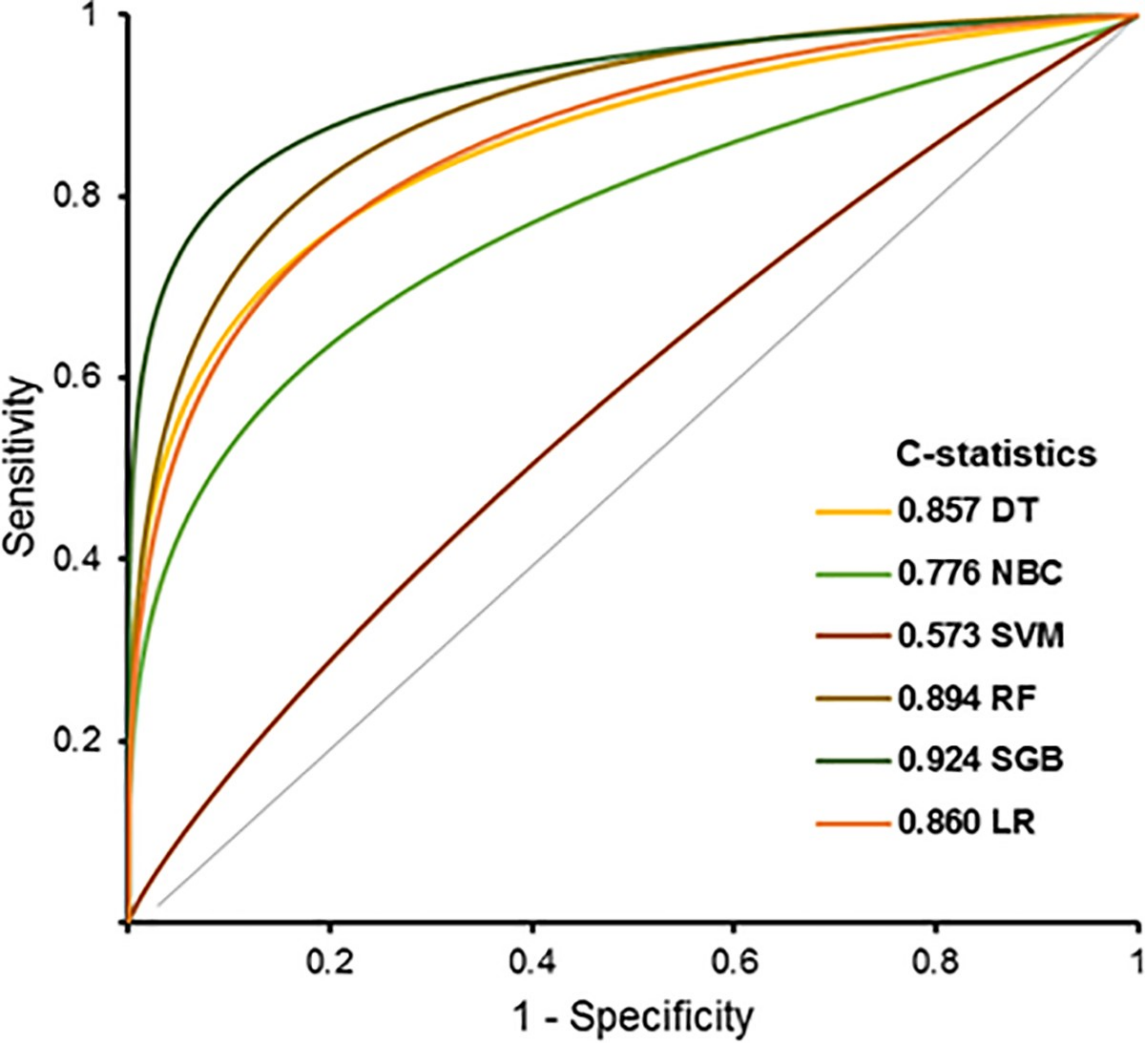
Receiver operating characteristic curves of late-onset preeclampsia prediction models. C-statistics for each prediction model are presented in the graph. ***Abbreviation*:** DT, decision tree; NBC, naïve Bayes classification; SVM, support vector machine; RF, random forest; SGB, stochastic gradient boosting; LR, logistic regression.

Calibration plots with respective C-statistics of DT, NB, SVM, RF, SGB, and LR models for predicting preeclampsia are shown in Fig 3. Notably, the C-statistics value model for predicting preeclampsia was highest in the SGB model, showing a value of 0.924. The C-statistics values for each of the DT, NB, SVM, RF, and LR were 0.857, 0.776, 0.573, 0.894, and 0.860, respectively. When the prediction performances were compared among the prediction models, the SGB model had the best performance for predicting preeclampsia. The overall accuracy of the SGB model was 0.973, false positive rate was 0.009, and detection rate reached 0.771 (Table 3).

**Table 3:** Comparison of prediction performances for late-onset preeclampsia development.

Models	Accuracy	Sensitivity	Specificity	Detection Rate
LR	0.862	0.703	0.870	0.209
DT	0.874	0.648	0.885	0.215
NBC	0.899	0.500	0.918	0.229
SVM	0.892	0.137	0.928	0.085
RF	0.923	0.679	0.935	0.336
SGB	0.973	0.603	0.991	0.771

***Abbreviation*:** LR, logistic regression; DT, decision tree; NBC, naïve Bayes classification; SVM, support vector machine; RF, random forest; SGB, stochastic gradient boosting

## Discussion

In this study, use of ML algorithms resulted in improved prediction performance of preeclampsia development compared to traditional statistical models. The accuracy and detection rate of the SGB model was superior to other prediction algorithms. In addition, influential variables for predicting preeclampsia were delineated which included several novel parameters.

The development of easy-to-use preeclampsia prediction methods has been a challenging subject. In this study, although the 2nd trimester characteristics did show statistically different values between the preeclampsia developing and non-developing group, the differences were minimal and not clinically noticeable. These clinically similar 2nd trimester characteristics are one of the main reasons that it is practically difficult to distinguish those who would develop preeclampsia from those who would not at this early time point of pregnancy. The fact that the pathogenesis of preeclampsia is complex and involves heterogeneous factors is one of the main causes of this difficulty [26,27]. Nonetheless, repeated attempts have been made to efficiently predict preeclampsia, which would lead to its early detection and prompt management. Identifying risk factors has been the most frequent approach to increase disease predictability. A previous history of preeclampsia, known chronic kidney disease, hypertension, diabetes, autoimmune disorders including systemic lupus erythematous and anti-phospholipid syndrome, maternal age > 40 years, and a body mass index > 35 kg/m^2^ are factors that have been reported to be associated with an increased preeclampsia development rate [28–31]. However, preeclampsia often occurs even in women without these risk factors, and additional strategies for its effective screening are limited. Several biomarkers have been also proposed to supplement the screening process for preeclampsia [32,33]. However, even with the help of these biomarkers, only 30% of cases of preeclampsia are predicted in advance [34]. The prediction model in this study effectively predicted the development of preeclampsia using demographic factors and antenatal laboratory data, which can be easily obtained in regular clinical practice. Even without the supplementation of biomarkers, the overall accuracy of the SGB model in this study was relatively high with a false positive rate of only 0.006. Therefore, the ML-based model proposed in this study could be used as a practical preeclampsia screening method during the antenatal period.

Several novel factors were found to impact preeclampsia prediction. Parameters that have been traditionally reported to be related to preeclampsia development such as BP, white blood cell count, creatinine level, liver function, and urinary protein were also determined to be influential factors in preeclampsia prediction. Interestingly, serum potassium levels were among the most important factors related with preeclampsia development. Although not thoroughly investigated, the relationship between serum potassium levels and hypertensive disorders during pregnancy has been often recognized. In a recent observational study of 8,114 deliveries, serum potassium levels during the first half of pregnancy was associated with a higher risk of severe preeclampsia [35]. Potassium homeostasis during pregnancy is affected by the activities of aldosterone and progesterone, both of which are known to play key roles in systemic vasodilatation [36]. Therefore, elevated serum potassium levels in pregnant women may be a surrogate for aldosterone and progesterone derangement, which could in turn be correlated with preeclampsia development. Serum calcium and magnesium levels were also closely associated with preeclampsia development [37]. This relationship has been proposed in several previous studies. Although controversial, low serum calcium levels during the antenatal period have been noticed in preeclampsia patients [38]. In addition, plasma magnesium levels were recently found to be higher in cases of mild and severe preeclampsia than in normal pregnancies [39]. The fact that calcium and magnesium play key roles in vascular smooth muscle constriction could explain this relationship.

The variables included in the prediction model were identified through pattern recognition. Pattern recognition and clustering was performed for repeated measured variables of regular antenatal evaluations preformed from early second trimester to 34 weeks’ of gestation. Previous studies investigating the relationship of clinical variables and preeclampsia development mostly used the mean value of a variable during a certain period. These investigations did not account for the fluctuation variability of the values. Recently, not only the mean value but also the fluctuation variability of a biomedical parameter has been suggested to have important clinical implications. Increased fluctuations in body weight and BP were found to increase the risk of cardiovascular diseases, while high variability in serum glucose levels were correlated with increased retinopathy risk in diabetic patients [40–42]. By incorporating the repeated measured values of the variables during the early second and third trimester period in the pattern recognition analysis, the value of the parameter itself as well as the changing pattern during the evaluation period was included as an analyzable factor. The pattern recognition and cluster analysis used with time series data allows the utilization of multiple aspects of a variable. In addition to using each value of the variables at different time points of a continuous time line, the changing patterns of the variables during the repeated measurement period could also be considered as a meaningful factor. This permits variables to be distinguished even if the values of the two variables are the same at a given point in time, as long as the pattern of change in the values in the continuous measurement is different. Such distinction would be important in interpreting repeated measured biological data. In a continuously increasing situation against a steadily decreasing situation, the same test value would undoubtedly have different clinical significance. This capability has enabled the successful use of pattern recognition to explore and exploit not only high-throughput measurement data [43], but also clinical data. Several recent investigations have used pattern recognition analysis for predicting adverse outcomes in chronic diseases [44–46]. In addition, unlike most of the evaluations assessing the association between biomarkers and preeclampsia development in which a hypothesis-based approach is used, a more objective and data-centric approach was possible by the application of pattern recognition. It should be noted that such an analytical approach has not been used before to predict preeclampsia in a large cohort of pregnant women.

This study has several limitations. First, most of the women were not included in the antenatal evaluation program until early second trimester. Therefore, first-trimester data could not be obtained. Although some reports have shown that early maternal changes were noticeable in women who develop preeclampsia [47]. most previous investigations have reported significant changes in the second and third trimesters. In addition, even without including first-trimester data, the predictive power of the SGB model was adequate. Second, the number of preeclampsia events was relatively smaller than in the control group. Nonetheless, considering the fact that the incidence of preeclampsia is 5–8% of all pregnancies, the number of preeclampsia cases was suitable considering the total study sample size [1]. In addition, the number of patients included in the present study was still larger than those of previous reports evaluating the association between clinical markers and preeclampsia development. Third, although antenatal evaluations were performed following the common protocol of our maternity care center, the evaluation intervals varied based on the participants’ symptoms and conditions. This could have influenced the prediction models. Nonetheless, since normal antenatal evaluations would be performed in a similar manner, the results of this study could have an advantage in being applied to real-world practice environments.

## Conclusions

The combination of maternal factors and common antenatal laboratory data from the early second and early third trimester using ML algorithms could effectively predict late-onset preeclampsia. Such algorithms could be applied in routine antenatal care to improve maternal and fetal outcomes of preeclampsia. Future studies prospectively verifying the accuracy of the proposed prediction algorithms are needed.

## Supporting information

S1 Table Importance of the selected variables for late-onset preeclampsia prediction models. (DOCX)

S1 Data Data set files used for the study analysis. (XLSX)
