# Supplementary material for: Prediction model development of late-onset preeclampsia using machine learning-based methods
Source: PLoS One. 2019 Aug 23;14(8):e0221202. doi: 10.1371/journal.pone.0221202 (PMC6707607; doi:10.1371/journal.pone.0221202)
Supplement: S1 Table — (DOCX) [file pone.0221202.s001.docx]

**Supporting information**

**S1 Table.** Importance of the selected variables for late-onset preeclampsia prediction models

| **Variables** | LR | DT | NBC | SVM | RF | SGB |
| --- | --- | --- | --- | --- | --- | --- |
| SBP | 0.65 | 1.00 | 0.99 | 0.20 | 0.70 | 1.00 |
| BUN | 1.00 | 0.62 | 0.99 | 0.03 | 0.80 | 0.69 |
| Creatinine | 0.02 | 0.08 | 1.00 | 0.54 | 0.45 | 0.57 |
| Platelet | 0.17 | 0.28 | 0.99 | 0.45 | 0.31 | 0.42 |
| Potassium | 0.03 | 0.28 | 1.00 | 0.28 | 0.36 | 0.34 |
| Calcium | 0.03 | 0.35 | 0.99 | 0.16 | 0.24 | 0.30 |
| UPCR | 0.08 | 0.27 | 0.99 | 0.35 | 0.14 | 0.21 |
| AST | 0.01 | 0.03 | 1.00 | 0.47 | 0.29 | 0.21 |
| TCO2 | 0.02 | 0.47 | 1.00 | 0.47 | 0.11 | 0.18 |
| WBC | 0.01 | 0.22 | 1.00 | 0.53 | 0.12 | 0.17 |
| ALT | 0.00 | 0.09 | 1.00 | 0.27 | 0.14 | 0.13 |
| Magnesium | 0.01 | 0.22 | 1.00 | 0.12 | 0.05 | 0.11 |
| UACR | 0.00 | 0.01 | 0.99 | 1.00 | 0.03 | 0.10 |
| Absolute t-score was used for LR and 1-accuracy for model excluding the relevant variable in NBC. IncNodePurity was used to compute the importance in DT, SVM, RF, and SGB models. The values were presented as original importance per maximum of the importance.  ***Abbreviation:*** LR, logistic regression; DT, decision tree; NBC, naïve Bayes classification; SVM, support vector machine; RF, random forest; SGB, stochastic gradient boosting; SBP, systolic blood pressure; WBC, white blood cell; UPCR, urine protein to creatinine ratio; UACT, urine albumin to creatinine ratio | | | | | | |
